# Prioritization Of Nonsynonymous Single Nucleotide Variants For Exome Sequencing Studies Via Integrative Learning On Multiple Genomic Data

**DOI:** 10.1038/srep14955

**Published:** 2015-10-13

**Authors:** Mengmeng Wu, Jiaxin Wu, Ting Chen, Rui Jiang

**Affiliations:** 1 MOE Key Laboratory of Bioinformatics; Bioinformatics Division and Center for Synthetic & Systems Biology, TNLIST; Department of Automation, Tsinghua University, Beijing 100084, China; 2 Department of Computer Science, Tsinghua University, China; 3 Molecular and Computational Biology Program, University of Southern California, USA; 4 Department of Statistics, Stanford University, 390 Serra Mall, Stanford, CA 94305-4065, USA

## Abstract

The rapid advancement of next generation sequencing technology has greatly accelerated the progress for understanding human inherited diseases via such innovations as exome sequencing. Nevertheless, the identification of causative variants from sequencing data remains a great challenge. Traditional statistical genetics approaches such as linkage analysis and association studies have limited power in analyzing exome sequencing data, while relying on simply filtration strategies and predicted functional implications of mutations to pinpoint pathogenic variants are prone to produce false positives. To overcome these limitations, we herein propose a supervised learning approach, termed snvForest, to prioritize candidate nonsynonymous single nucleotide variants for a specific type of disease by integrating 11 functional scores at the variant level and 8 association scores at the gene level. We conduct a series of large-scale *in silico* validation experiments, demonstrating the effectiveness of snvForest across 2,511 diseases of different inheritance styles and the superiority of our approach over two state-of-the-art methods. We further apply snvForest to three real exome sequencing data sets of epileptic encephalophathies and intellectual disability to show the ability of our approach to identify causative *de novo* mutations for these complex diseases. The online service and standalone software of snvForest are found at http://bioinfo.au.tsinghua.edu.cn/jianglab/snvforest.

Recently, next-generation sequencing technology has been successfully applied in both genetic and genomic studies, as demonstrated in such breakthrough events as the production of a public catalog of human genetic variation^1^ and the identification of functional elements in the human genome^2^, among many others^3^,^4^,^5^,^6^. Particularly, exome sequencing, as an efficient technique for large-scale capture of genetic variants in protein-coding regions, has been demonstrated as an effective way for the detection of pathogenic variants for both Mendelian diseases^7^ and complex diseases^8^.

A hallmark of exome sequencing is the ability to identify rare nonsynonymous single nucleotide variants (nsSNV), which occur in low allele frequency (≤1%) and are of particular interest for the discovery of novel disease-causing variants. In a typical workflow of analyzing exome sequencing data, candidate rare variants are obtained by applying bioinformatic tools as ANNOVAR^9^ to annotate variants identified from a cohort, resorting to resources as the 1000 Genomes Project to discard variants of high frequency, and relying on databases as dbSNP^10^ to filter out variants present in healthy individuals. In addition, computational approaches such as SIFT^11^ and PolyPhen2^12^ are often used to predict functional implications of variants, for the purpose of screening out candidates that are likely to be functionally neutral. To further increase the power of such filtration, a variety of methods have also been proposed to integrate multiple deleterious scores of variants^13^,^14^, resulting in such comprehensive data repositories as dbNSFP^15^ for whole-exome functional predictions of nonsynonymous variants. Nevertheless, reliance on predicted functional implications to infer disease-causing variants usually results in high false positive rates, probably because susceptible variants may be only mildly deleterious, and more importantly, predicted functionally damaging effects are not specific to the disease of interest. For example, in the Swiss-Prot database^16^ (release 2014_01), about 10.94% nonsynonymous variants annotated as “Disease” are predicted benign by PolyPhen2, and a total of 14,691 variants assigned the most extreme deleterious scores by SIFT are known to be causative for a total of 1,896 diseases.

Computational efforts for the prioritization of candidate genes provide a means of connecting genes to a specific type of disease. Taking advantage of the guilt-by-association principle^17^ and a variety of genomic data sources such as the gene expression^18^, protein-protein interaction^19^, gene ontology^20^, and many others^21^,^22^, genes potentially causative for a query disease can be inferred to provide the guidance for subsequent functional test. However, the association between a gene and a disease does not necessarily imply that every variants in the gene is causative for that disease. For example, about 12.62% genes hosting causative variants also contain neutral variants according to the Swiss-Prot database^16^.

To overcome respective limitations of the above two categories of approaches, studies have been performed to integrate functionally damaging effects at the variant level and association information at the gene level for more accurate inference of causative variants. For example, Sifrim *et al.* proposed a method called eXtasy^23^ that combined 7 types of variant functional prediction scores, 2 types of gene association scores and several disease phenotype-related scores through Endevour^24^ to prioritize candidate nonsynonymous variants. Wu *et al.* developed a method named SPRING^25^ that integrated 6 types of variant scores and 5 types of gene scores with a rigorous statistical model to predict disease-causing variants. Nonetheless, even though these methods did improve the accuracy of inferring pathogenic variants in exome sequencing studies, they also suffered from their respective limitations. For example, eXtasy requires genes known as associated with HPO (Human Phenotype Ontology) terms, thus limiting its application for diseases without accurate HPO annotations. SPRING does not use any positive sample at all and thus may waste prior knowledge collected in such databases as OMIM^26^ and HGMD^27^.

To overcome the limitations of these methods, we propose a bioinformatics approach called snvForest for the prioritization of nonsynonymous single nucleotide variants. Our method takes a query disease and a set of candidate variants as input, predicts the strength of associations between the candidates and the query disease, and produces a ranking list of the candidates as output. Specifically, we achieve this goal by adopting an ensemble learning method named the random forest^28^ to integrate 11 scores that assess the functionally damaging effects of the candidate variants and 8 scores that evaluate the strength of associations between the query disease and the genes hosting the variants. We perform a series of large-scale validation experiments to demonstrate the effectiveness of snvForest across 2,511 diseases of different inheritance styles and its superiority over two existing state-of-the-art approaches. We further applied snvForest to three real exome sequencing datasets for epileptic encephalopathies and intellectual disability to show the ability of our method to identify causative *de novo* mutations for these complex diseases. We provide the online service and the standalone software of snvForest at http://bioinfo.au.tsinghua.edu.cn/jianglab/snvforest.

## Results

### Overview of snvForest

**Figure 1:**
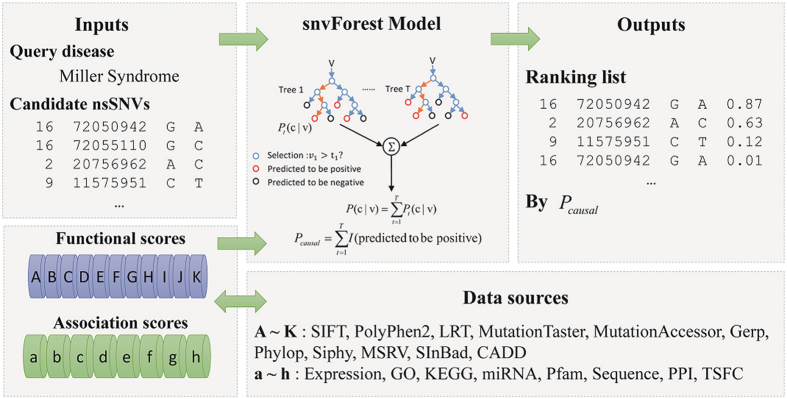
Overview of snvForest. Taking a query disease and a set of candidate nonsynonymous single nucleotide variants (nsSNVs) as input, snvForest predicts the strength of associations between the candidates and the query disease, and produces a ranking list of the candidates as output. We achieve this goal by adopting an ensemble learning method named the random forest to integrate 11 functional scores that assess the functionally damaging effects of the candidate variants and 8 association scores that evaluate the strength of associations between the genes hosting the variants and the query disease.

We based the design of our method, termed snvForest, on the notion that the inference of disease-causing nonsynonymous single nucleotide variants (nsSNVs) should be made through the integration of genomic information at both variant and gene levels. As illustrated in Fig. 1, our method took a query disease and a set of candidate variants as inputs and produced a ranking list of the candidates as output, with variants more likely to be causative for the query disease ranked higher. To accomplish this, we adopted a supervised learning method called the random forest to predict the strength of associations between the candidate variants and the query disease based on 11 functional scores at the variant level and 8 association scores at the gene level.

More specifically, the 11 functional scores predicted the likelihood that a variant would damage the function of its hosting gene, and these scores were calculated by such bioinformatics approaches as SIFT^11^, PolyPhen2^12^, LRT^29^, MutationTaster^30^, MutationAccessor^31^, MSRV^32^, GERP^33^, Phylop^34^, SiPhy^35^, CADD^36^ and SInBaD^37^. The 8 association scores measured the likelihood that a gene containing a variant would associate with a query disease, and such scores were derived according to the “guilt-by-association” principle^17^ from such genomic data as gene expression (Exp)^18^, gene ontology (GO)^20^, KEGG pathway (KEGG)^21^, protein sequence (Seq)^38^, protein domain (Pfam)^22^, protein-protein interaction (PPI)^19^, transcriptional regulation (TSFC)^39^, and microRNA regulation (miRNA)^40^. As detailed in Methods, from each type of the genomic data, we derived a gene functional similarity matrix. From the OMIM and UMLS databases, we derived a disease phenotype similarity matrix. To characterize the strength of association between a gene and a query disease under a type of genomic data, we made use of the phenotype similarity matrix to identify a set of seed genes for the query disease and then calculated the summation of similarities between the seed genes and the given gene. The coverage of the above data sources is shown in the Supplementary Materials (Table S1).

With the information at both variant and gene levels ready, we modeled the prioritization problem as a procedure of predicting strength of associations between the candidate variants and the query disease. Specifically, we regarded a disease-variant pair as belonging to either a positive class, indicating that the variant is causative for the disease, or a negative category, indicating that the variant is not relevant to the disease. We then adopted the random forest approach to predict the likelihood that the disease-variant pair belonged to the positive class, thereby providing a means of prioritizing the candidate variants.

### Performance in cross-validation experiments

We customized a five-fold cross-validation experiment to assess the effectiveness of our approach in distinguishing disease-causing variants from irrelevant ones. From the Swiss-Prot database, we collected 25,559 disease variants that were causative for 2,511 diseases and 38,910 neutral variants that were irrelevant to diseases. We then split the disease variants into five subsets of almost equal size according to their associated diseases and the neutral variants into two subsets of equal size (19,455 variants), one for training and the other for test. In each fold of the validation, we took a disease subset as the positive test sample and a neutral subset as the negative test sample, trained an snvForest model using variants in the remaining subsets, and prioritized each variant in the positive test sample against those in the negative. In order to avoid information leakage^41^, we took two extra steps to eliminate potential overlaps between genes involved in the training and test phases. First, we removed seed genes that were associated with any test diseases or hosted any test variants. Second, we discarded training variants that occurred in the same gene as any test variants. By doing this, we made certain that no information about diseases, genes and variants could be shared between training and test data, thus eliminated the possibility of overfitting and made the validation procedure unbiased.

**Figure 2:**
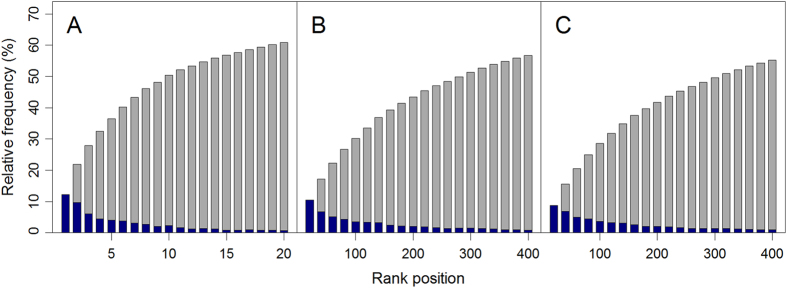
Ranking performance of snvForest. Bars are probability mass (blue) and cumulative distribution (gray) of positive test variants ranked among top 20 and top 400 in the cross-validation experiment against the (**A**) neutral test sample, (**B**) HGMD test sample, (**C**) combined test sample.

Repeating the validation 5 times until every disease subset served as the test sample once, we collected the results and summarized rank positions of positive test variants in Fig. 2(A), which clearly highlights the effectiveness of our method in pinpointing disease-causing variants in a pool of neutral candidates. For example, our method ranks 3,136 (12.27%) disease variants first, 12,881 (50.40%) among top 10, and 15,545 (60.82%) among top 20, respectively. In contrast, a random guess procedure on average can only rank 25,559/(19,455 + 1) ≈ 1.32 disease variants first, 13.15 among top 10, and 26.30 among top 20. The capability of our approach in discriminating causative variants from neutral ones is therefore strongly supported.

We then assessed the effectiveness of our method in distinguishing variants causative for a query disease from those associated with other diseases. For this purpose, we repeated the cross-validation experiment with the negative test sample replaced by a set of 20,000 variants that were collected from the HGMD database. Results, as shown in Fig. 2(B), also demonstrated the effectiveness of our method in this situation. For example, our method ranks 1,541 (6.03%) and 2,671 (10.45%) positive test variants among top 10 and top 20, respectively. Considering that a random guess procedure can only rank 10 × 25,585/(20,000 + 1) ≈ 12.79 positive test variants among top 10 and 25.58 among top 20, the capability of our approach in discriminating variants for a query disease from those relevant to other diseases is also strongly supported.

We also simulated the situation that an individual carried not only neutral variants but also some disease variants relevant to some diseases by repeating the cross-validation experiment using the combination of the neutral and HGMD variants as the negative test sample. Results, as shown in Fig. 2(C), again strongly support the capability of our approach in this situation by ranking 1,178 (4.61%) and 2,217 (8.67%) positive test variants among top 10 and top 20, respectively.

We further derived two comprehensive criteria to evaluate the performance of our method. We defined the rank ratio of a variant as its rank divided by the total number of candidates in a list. Averaging rank ratios over test variants associated with a disease and further averaging the resulting quantities over all diseases, we obtained a criterion called the mean rank ratio (MRR). For a specific type of disease, we identified its associated positive test variants, corresponding ranking lists, and distinct rank ratio values in these lists. Taking each of such values as a threshold and focusing on the ranking lists corresponding to the disease, we calculated the sensitivity at a threshold as the fraction of positive test variants whose rank ratios were less than or equal to the threshold and the specificity as the fraction of negative test variants whose rank ratios exceeded the threshold. Varying the threshold value from the smallest to the largest, we drew the rank receiver operating characteristic (ROC) curve (sensitivity versus 1-specificity) and calculated the area under this curve. Averaging such area values over all diseases, we obtained a criterion called the mean area under the ROC curve (AUC). As summarized in Table 1, in the validations against the neutral, HGMD and combined test samples, our method achieves MRRs of 3.37%, 15.97% and 9.76%, respectively and AUCs of 96.60%, 84.00% and 90.23%, respectively. Considering that a random guess procedure could only yield an MRR of 50% and an AUC of 50%, the effectiveness of our method is evident. We further analyzed distributions of these two evaluation criteria for individual diseases and showed that our method could be effectively applied to most diseases (Supplementary Materials, Figure S1).

### Superiority over existing methods

We compared the performance of our method with two existing state-of-the-art methods for prioritizing candidate nonsynonymous SNVs, eXtasy^23^ and SPRING^25^. Using the standalone software of these methods provided in their official websites, we found that snvForest, and SPRING could cover all the 25,559 variants in the Swiss-Prot database, while eXtasy could only cover 19,742 (77.16%) of these variants. Therefore, snvForest and SPRING are superior to eXtasy in terms of the coverage of variants.

**Figure 3:**
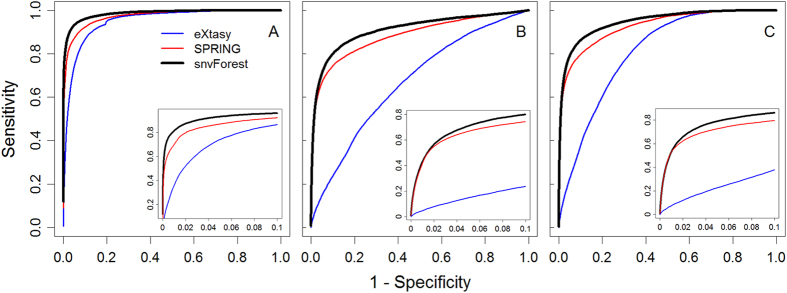
Superiority over existing methods. Rank receiver operating characteristic curves (ROCs) of snvForest, eXtasy and SPRING in validation experiments against (**A**) neutral, (**B**) HGMD and (**C**) combined test samples. Curves are drawn by pooling the validation results for all the 2,511 diseases and those “plots within plots” are zoomed-in regions of AUC curves. The curves of snvForest and SPRING both stay above that of eXtasy, indicating the higher ability of the former two methods to enrich true causative variants among top rank positions.

We then focused on the variants coveraged by all the three methods and used the aforementioned validation procedure to compare their performance. As summarized in Table 1, in the validation against the neutral test sample, snvForest achieves an MRR of 3.26%, outperforms those of eXtasy (7.77%) and SPRING (4.15%). In the validation against the HGMD test sample, snvForest achieves an MRR of 16.02%, also outperforms those of eXtasy (43.48%) and SPRING (17.25%). In the validation against the combined test sample, snvForest achieves an MRR of 9.64%, again outperforms those of eXtasy (25.87%) and SPRING (10.79%). Pairwise one-sided Wilcoxon rank sum tests also show that median rank ratios of positive test variants against the three negative test samples for snvForest are significantly less than those for SPRING, which are in turn less than those of eXtasy (Supplementary Materials, Table S2). Besides, in all the three validation experiments, the AUCs for snvForest are higher than those of SPRING, which are in turn higher than those of eXtasy. We further pooled test variants for all the 2,511 diseases and plotted overall ROC curves of the three methods in Fig. 3. From the figure, we clearly see that the curves of snvForest and SPRING both stay above that of eXtasy, indicating the higher ability of the former two methods to enrich true causative variants among top rank positions. All these results suggest that snvForest is superior to SPRING, and both of them are superior to eXtasy in terms of the prioritization performance.

We analyzed that the superiority of our method over eXtasy and SPRING may be due to the following reasons. First, SPRING adopted Fisher’s method to integrate multiple genomic data sources. An advantage of this approach is that it does not rely on known causative variants. However, this could also be a drawback in the sense that making use of known positive information may improve the performance. Second, eXtasy trained models on phenotype-variant combination. Therefore, this method might be less effective when the disease under investigation is described by some general HPO terms that are also used to describe other diseases. In contrast to these two methods, our approach makes use of known positive information, thereby overcoming the disadvantage of SPRING. Our approach trains model on disease-variant combination, thereby overcoming the disadvantage of eXtasy. As a result, our approach outperforms both SPRING and eXtasy in the above validation experiments, in which we aim to identify causal variants for a given disease. Therefore, our method is more suitable for the application to an exome sequencing study on a specific type of disease (e.g. a disease from OMIM), while eXtasy is more suitable for undiagnosed diseases with HPO annotations.

We further compared the performance of our approach with five other supervised learning methods, including linear discriminant analysis (LDA), naïve Bayes (NB), logistic regression (LR) and support vector machine (SVM). Results suggest that the performance of these methods can be ordered from the highest to the lowest as snvForest > SVM > LR > NB > LDA (Supplementary Materials, Table S3).

### Performance for diseases of different inheritance styles

We assessed the effectiveness of our method for diseases of different inheritance styles based on the cross-validation results obtained previously. For a category of inheritance style, we identified diseases belonging to this category and averaged the values of an evaluation criterion over such diseases to evaluate the performance of our method for this inheritance style.

**Figure 4:**
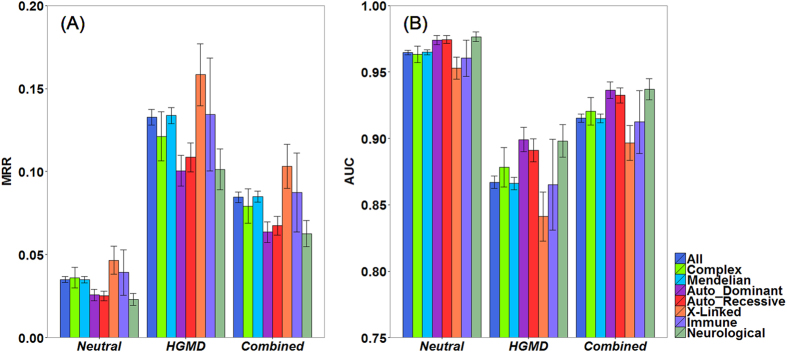
Performance for diseases of different inheritance styles. (**A**) MRRs and (**B**) AUCs of snvForest for different categories of inheritance styles against the three negative test samples. Diseases are classified into eight categories according to their inheritance styles. Based on the previous cross-validation results, the performance of our method for a category is calculated by identifying diseases belonging to this category and averaging the values of an evaluation criterion (MRR or AUC) over such diseases. Error bars denote the standard errors of corresponding quantities.

We first categorized the 2,511 diseases into a group of 189 complex diseases and a group of 2,322 Mendelian diseases according to the Genetic Association Database^42^. Results, as shown in Fig. 4, suggest that our method is effective for both categories of diseases. For example, in the validation against the neutral control, the MRR is 3.61% for complex diseases and 3.49% for Mendelian diseases. A two-sided Wilcoxon rank sum test suggests that the median rank ratios of test variants for these two groups of diseases are not significantly different (*p*-value = 0.3064 with Bonfferoni correction).

We then categorized the 2,322 Mendelian diseases into three groups with different inheritance patterns, including 455 autosomal dominant diseases (MIM: 1xxxxx), 537 autosomal recessive diseases (MIM: 2xxxxx) and 184 X-Linked diseases (MIM: 3xxxxx). Results, as shown in Fig. 4, again suggest that our method is effective for all these types of diseases. For example, in the validation against the neutral control, our method achieves MRRs of 2.57%, 2.51% and 4.66% for autosomal dominant, autosomal recessive and X-linked diseases respectively. Two-sided wilcoxon tests suggest that the performance of our method is slightly different between autosomal dominant and autosomal recessive diseases (*p*-value = 0.0455 with Bonfferoni correction), not different between autosomal dominant and x-linked diseases (*p*-value = 0.0650 with Bonfferoni correction), and not different between autosomal recessive and x-linked diseases (*p*-value = 1 with Bonfferoni correction).

We finally evaluated the performance of our method for 47 immune diseases and 256 neurological diseases, and we also found that our method is also effective for both types of diseases (Fig. 4). For example, in the validation against the neutral control, the MRRs are 3.92% and 2.29% for immune and neurological diseases, respectively. However, a two-sided Wilcoxon test does not support difference between these two types of diseases (*p*-value = 0.8715 with Bonfferoni correction).

### Performance for rare variants

Rare variants occur in very low frequency (<1%) in a population and are believed to be involved in biological processes of complex diseases^43^. We therefore evaluated the effectiveness of our method in identifying causative rare variants. Focusing on the Swiss-Prot database, we extracted 895 rare causative variants with minor allele frequency 1% or less according to dbSNP and identified 533 associated diseases. We then trained an snvForest model by using variants associated with the remaining 1,978 diseases as positive training data and half of the neutral variants as negative training data, with overlapping information between the training and test phases eliminated using the aforementioned strategy. Finally, we prioritized each of the 895 rare variants against the three negative test samples defined previously.

Results show that our method is effective in identifying disease-causing rare variants. For example, in the validation against the neutral control (19,455 variants), our method ranks 362 out of the 895 variants among top 20. In contrast with a random guess procedure that can only enrich 895 × 20/19,455 ≈ 0.92 variants among top 20, the effectiveness of our method is strongly supported. In the validation against the disease and combined control, our method ranks 52 and 47 variants among top 20, respectively. Overall, when validating against the neutral, disease and combined controls, our method achieves MRRs of 6.24%, 19.14% and 12.78%, respectively, and AUCs of 93.75%, 80.85% and 87.22%, respectively. All these observations support the effectiveness of our method in identifying disease-causing rare variants. We also observed that snvForest owned the best performance when compared with the other methods (Supplementary Materials, Table S4).

### Importance of individual features

**Figure 5:**
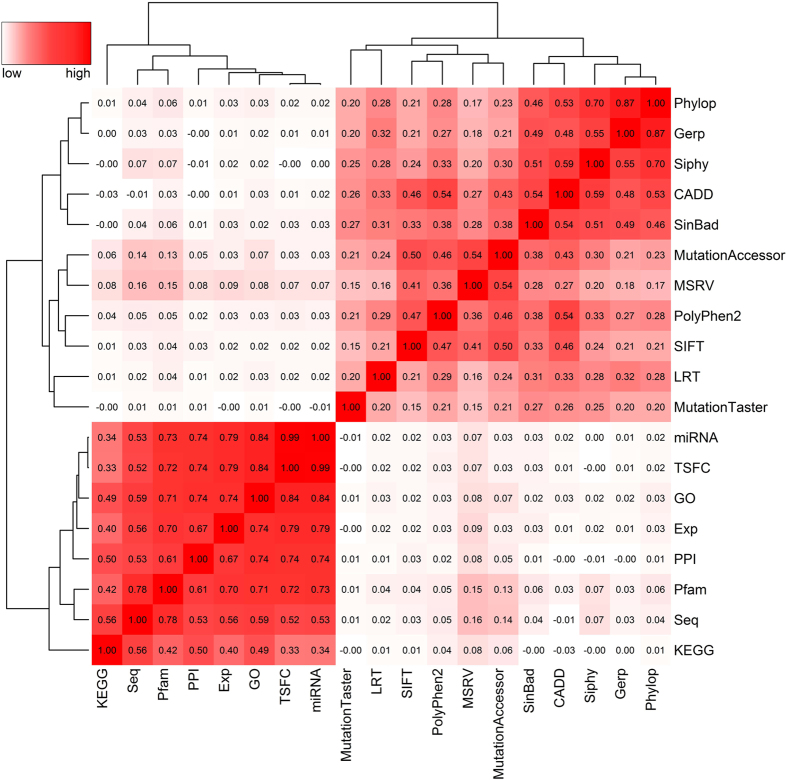
Pairwise Pearson’s correlation coefficients between the features based on disease variants. There exist low to strong positive correlations among the 8 association scores at the gene level (bottom-left region). There exist medium to strong positive correlations among the 11 functional scores at the variant level (top-right region). The correlations between functional scores and association scores are weak.

The 19 features used by our method can be classified to a group of 11 functional scores at the variant level and a group of 8 association scores at the gene level. We first focused on the 25,559 disease variants in the Swiss-Prot database to calculate pairwise Pearson’s correlation coefficients between the features and performed a hierarchical cluster analysis on the resulting matrix. Results, as illustrated in Fig. 5, clearly show three patterns. First, there exist low to strong positive correlations among the 8 association scores at the gene level (bottom-left region). Second, there exist medium to strong positive correlations among the 11 functional scores at the variant level (top-right region). Particularly, the five features relying on the multiple sequence alignment of DNA sequences (Phylop, Gerp, Siphy, CADD and SInBaD) are highly correlated with each other, and so do the four features based on the alignment of protein sequences (MutationAccessor, MSRV, PolyPhen2 and SIFT). Third, the correlations between functional scores and association scores are weak, since those two types of features characterize different aspects of a disease variant. We then focused on neutral variants to calculate pairwise Pearson’s correlation coefficients of the features. Result showed that correlations between the association scores are much weaker than those calculated according to disease variants, revealing that similarities between the gene hosting a neutral variant and seed genes of a query disease exhibit diversity among different data sources (Supplementary Materials, Figure S2).

We then assessed the performance of each of the 19 features by repeating the validation experiments with the use of a single feature and summarized the results in Table 2. We first observe from this table the effectiveness of features at the variant level in distinguishing disease variants from neutral ones, given the low MRRs (15.29% to 27.43%) and high AUCs (72.57% to 84.71%). We also observe the ineffectiveness of these features in discriminating between disease variants, considering that both MRRs and AUCs are around 50%. This phenomenon is due to the fact that all these features do not contain information that helps to identify the specific disease with which a variant is associated. Combining these two aspects, the variant features show low effectiveness in distinguishing causative variants from the combined negative test sample (MRRs ranging from 29.76% to 40.42% and AUCs ranging from 59.58% to 70.24%). The above conclusions are further supported by the observation that distributions of functional scores for disease variants are significantly different from those for neutral variants, while these scores are not differentially distributed for disease variants in Swiss-Prot and HGMD (Supplementary Materials, Figure S3).

Table 2 also shows that features at the gene level exhibit medium effectiveness in distinguishing disease variants from neutral, disease and combined negative test samples. Specifically, the MRRs for these three types of validation experiments range from 19.78% to 40.90%, 25.55% to 41.95% and 22.94% to 41.42%, respectively, while the AUCs range from 59.10% to 80.22%, 58.05% to 74.45% and 58.58% to 77.06%, respectively. These observations can be explained as follows. The association score assigned to a variant is calculated based on its host gene, and hence variants will be assigned identical scores as long as they occur in the same gene. From the Supplementary Materials (Figure S4), we observe that distributions of association scores for disease variants in the Swiss-Prot database are different from those for both neutral variants in the Swiss-Prot database and variants in HGMD. Therefore the association scores have the ability to not only discriminate between neutral and disease variants but also distinguish disease variants causing a specific type of disease from those responsible for other diseases.

Finally, we notice that the integration of all the features yields a method with much higher performance than any individual feature. Specifically, the MRRs are 3.37%, 15.97% and 9.76% in the validation against neutral, disease and combined negative test samples, respectively, and the AUCs are 96.60%, 84.00% and 90.23%, respectively. Besides, the coverage of our method also benefits from data integration. For example, in the 1000 Genomes Project data, only 56.09% variants have PolyPhen2 scores, and the coverage of pathway data is even as low as 34.25%. We address the missing values by assigning zeros to them. With the integration of multiple data sources, however, the causative effect of a variant for a query disease can be predicted as long as the variant appears in a data source, and thus the coverage of our method is extended to the union of variants included in individual data sources.

### Performance in simulated exome sequencing studies

We performed a spike-in simulation experiment to assess the performance of snvForest in identifying disease variants in exome sequencing studies. To accomplish this, we extracted from the 1000 Genomes Project^1^ a total of 251,235 nonsynonymous variants occurring in 1,092 subjects. We found that on average a subject owned 9,512 nonsynonymous variants, consistent with existing studies^1^. By mapping annotated disease variants in the Swiss-Prot and HGMD databases into individual exomes, we also found that on average about 30 pathogenic variants are present in an exome, revealing the importance of distinguishing variants causative for a query disease against those responsible for other diseases (Supplementary Materials, Figure S5). With a similar validation strategy as described previously, we partitioned the 25,559 disease variants in the Swiss-Prot database into five positive subsets of nearly equal size according to their associated diseases. We then repeatedly used four positive subsets with all neutral variants in the Swiss-Prot database to train an snvForest model and prioritized each disease variant in the remaining positive subset against variants occurring in a subject of the 1000 Genomes Project. In this procedure, we also eliminated overlapping information between the training and test data using the aforementioned strategy.

**Figure 6:**
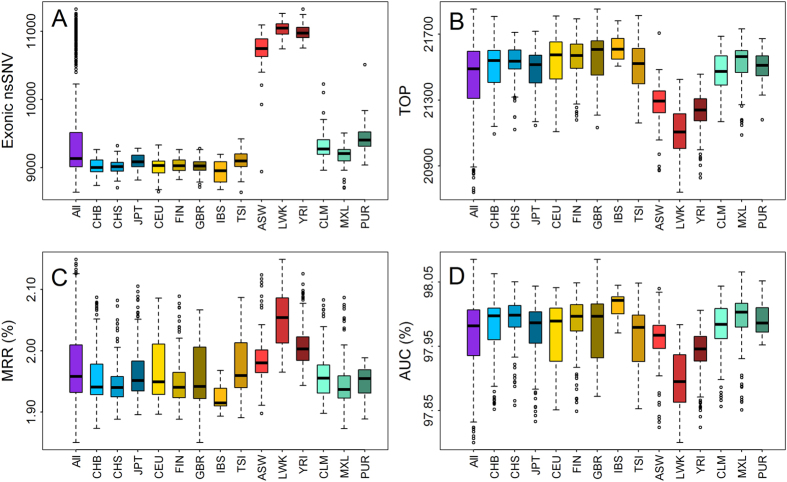
Performance on exomes derived from the 1000 genomes project. (**A**) Number of nonsynomous single nucleotide variants, (**B**) Number of disease-causing variants ranked among top 20, (**C**) MRRs and (**D**) AUCs for different populations. The x asix denotes different populations according to sample descriptions from 1000 Genome Project. Population abbreviations: ASW, people with African ancestry in Southwest United States; CEU, Utah residents with ancestry from Northern and Western Europe; CHB, Han Chinese in Beijing, China; CHS, Han Chinese South, China; CLM, Colombiansin Medellin, Colombia; FIN, Finnish in Finland; GBR, British from England and Scotland, UK; IBS, Iberian populations in Spain; LWK, Luhya in Webuye, Kenya; JPT, Japanese in Tokyo, Japan; MXL, people with Mexican ancestry in Los Angeles, California; PUR, Puerto Ricans in Puerto Rico; TSI, Toscani in Italia; YRI, Yoruba in Ibadan, Nigeria. Ancestry-based groups: AFR, African; AMR, Americas; EAS, East Asian; EUR, European.

Results, as illustrated in Fig. 6(B–D), show that the disease variants spiked in can be well distinguished. For example, on average 21,443 out of the 25,559 disease variants are enriched among top 20 for a subject, significantly higher than a random guess procedure that can only rank 25,585 × 20/(9,512 + 1) ≈ 53.79 variants among top 20. For the two evaluation criteria, snvForest achieves an MRR of 1.97% and an AUC of 97.90% for a subject, further suggesting the effectiveness of our method in exome sequencing studies. We also notice that the performance of snvForest for the three African-related populations (ASW, LWK and YRI) is slightly lower than that for the other populations, probably due to the fact that subjects in these three populations own more variants than do those from the other populations (Fig. 6(A)).

**Figure 7:**
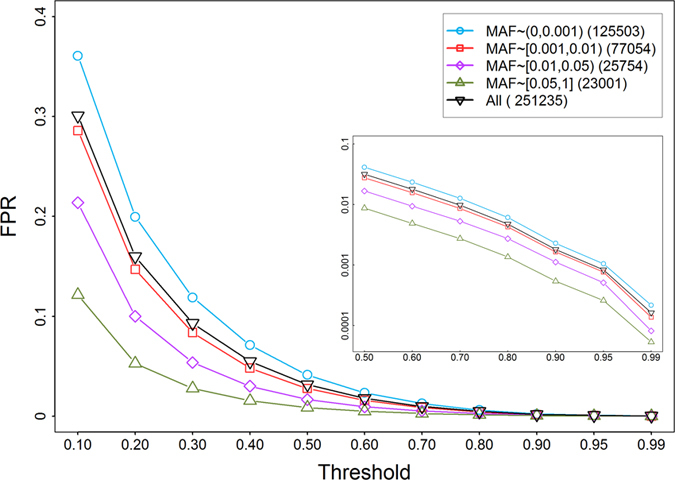
False positive rates estimated using exomes derived from the 1000 genomes project. The false positive rate for a given threshold is defined as the proportion of neutral nonsynomous single nucleotide variants that are present in the 1000 genomes project and whose prediction scores are greater than the threshold value. The numbers inside the brackets denote the number of SNVs falling into the corresponding MAF range.

The prediction score calculated by snvForest can be used in two ways. First, for a set of candidate variants, their scores can be used as bases for prioritization. Second, for a single variant, its score can be used to predict whether the variant is causative for a query disease. We therefore assessed false positive rates (FPR) at different levels of the prediction score. Specifically, we calculated prediction scores of all nonsynonymous variants in the 1000 Genomes Project for each of the 2,511 diseases, and estimated the FPR for a disease as the proportion of such neutral variants whose scores were higher than or equal to a threshold, and averaged over all diseases to obtain an estimate of the overall FPR. Results, as illustrated in Fig. 7, suggest that the FPR can be well controlled via the selection of a suitable threshold of the prediction score. For example, at the thresholds 0.8, 0.9, 0.95 and 0.99, the overall FPRs are 0.48%, 0.18%, 0.08% and 0.02%, respectively. We further estimated FPRs for variants of different minor allele frequencies (MAFs). Results show that the FPR increases with the decrease of the allele frequency at a certain threshold value, suggesting that the correct identification of rare disease variant is more difficult than that of common ones^43^.

### Applications to real exome sequencing studies

To demonstrate the effectiveness of snvForest in identifying causative mutations for complex diseases, we applied our method to three real exome sequencing datasets. Epileptic encephalopathies are among the most deleterious groups of childhood epilepsy disorders. In a recent study^44^, exome sequencing was applied to 264 probands with their parents, showing strong statistical evidence on the association of several *de novo* mutations with this group of complex diseases. From this study, we collected 192 unique candidate nonsynonymous *de novo* mutations, among which 30 were reported as likely functional in the literature^44^. With the criterion that a seed gene should not host any candidate mutations and should have been reported as associated with epileptic encephalopathies by independent studies before the publication of this data set, we collected from the OMIM database a total of 9 seed genes that contained none of the 192 candidate mutations. We then used all variants in the Swiss-Prot database to train a snvForest model and prioritized the 192 candidate mutations. To achieve an unbiased evaluation, we excluded from the training data the candidate variants, their host genes, variants occuring in these genes, and diseases with which these genes were associated. We also excluded genomic data sources that have been used in the original study.

Results (Table 3) show that 8 mutations ranked among top 10 are likely functional, revealing the capability of our method in uncovering causative variants for this disease. A one-sided Fisher’s exact test suggests that the probability of ranking 8 or more mutations among top 10 by chance is only 5.3 × 10^−6^, strongly supporting the power of our method. Furthermore, a one-sided Wilcoxon rank sum test suggests that the prediction scores of the reported 30 functional mutations are significantly larger than those of the other candidates (*p*-value = 1.64 × 10^−10^), supporting the effectiveness of our method. We further notice that only 3 mutations have prediction scores over 0.90, and all of them are reported causative in the literature^44^. Among mutations whose prediction scores are larger than 0.8, the 11^th^ one introduces a missense alteration on GNAO1, and this gene has been recently reported as involved in epileptic encephalopathies in an independent study^45^. These results further support the high predictive power of our method.

Intellectual disability is another deleterious complex disorder whose genetic causes remained largely unknown for its clinical and genetic heterogeneity. Many efforts were made to investigate the genetic basis of this disorder^46^,^47^. For example, De Ligt J *et al.* sequenced exomes of 100 patients whose IQs are below 50 and their unaffected parents and found 3 *de novo* mutations with strong evidence for causing intellectual disability besides the 10 *de novo* mutations and 3 X-linked mutations that were previously predicted to disrupt functions of known intellectual disability genes^46^. From this study, we collected 77 unique candidate nonsynonymous *de novo* mutations, among which 16 were reported to be likely functional. We used the same strategy as above to obtain 13 unbiased seed genes and a trained snvForest model, and we used the model to prioritize the candidate mutations.

Results (Table 3) show that the two mutations ranked first and second are both likely functional. A one-sided Fisher’s exact test suggests that the probability of ranking 2 causative mutations as first and second by chance is only 0.041, supporting the power of our method. Furthermore, 5 mutations ranked among top 10 are likely functional, yielding a Fisher’s exact test *p*-value of 0.028. A one-sided Wilcoxon rank sum test suggests that the prediction scores of the reported 16 functional mutations are significantly larger than those of the other candidates (*p*-value = 4.51 × 10^−3^), supporting the effectiveness of our method. Among those variants whose prediction scores are larger than 0.5, the mutation ranked at 16^th^ (a damaging mutation of EEF1A2) has been reported to be involved in this disorder in an independent study^48^.

Another study on intellectual disability was performed by Rauch *et al.*^47^, in which 51 children with intellectual disability from German Mental Retardation Network and their unaffected parents were treated as cases, while controls consisted of 20 healthy children along with their unaffected parents. We collected 126 *de novo* mutations as candidates from this study and manually selected 12 seed genes for unbiased evaluation. Results (Table 3) show that the two mutation ranked first and second are likely functional. A one-sided Fisher’s exact test suggests that the probability of ranking two causative mutations at first and second by chance is only 0.017, supporting the power of our method. Besides, 5 mutations ranked among top 10 are likely functional, yielding a Fisher’s exact test *p*-value of 4.1 × 10^−3^. A one-sided Wilcoxon rank sum test suggests that the prediction scores of the reported 16 functional mutations are significantly larger than those of the other candidates (*p*-value = 1.77 × 10^−5^), again supporting the effectiveness of our method. We observe that only 4 mutations are assigned prediction scores over 0.8, and 3 of them are reported as likely functional. A literature search further show that the one (a missense mutation in KCNH1) not reported as functional in the literature^47^ is recently reported as involved in Temple-Baraitser syndrome^49^, a multisystem developmental disorder leading to intellectual disability and other related similar phenotypes. These results further suggest the effectiveness of our method.

## Discussion

In this paper, we have proposed a bioinformatics approach called snvForest to prioritize candidate nonsynonymous SNVs. We demonstrate the superior performance of snvForest over two existing methods via a series of comprehensive validation experiments and further show the power of our method in the identification of causative *de novo* mutations in three real exome sequencing data.

The high performance of snvForest is due to a combination of the following aspects. First, we make use of not only functionally damaging effects of variants but also associations between genes hosting the variants and the query disease. The former contributes to the discrimination of disease variants against neutral ones, while the later helps to draw a distinction between variants causative for different types of diseases. As a result, our method gains the capability of identifying variants causative for a specific type of disease from candidate variants. Second, we adopt a powerful supervised learning method that uses both positive and negative samples to integrate multiple data sources. Consequently, snvForest achieves higher performance than existing state-of-the-art methods such as SPRING^25^ and eXtasy. In addition, data integration also helps our method to achieve high robustness and improve coverage.

The most important ingredient in the application of our method is the selection of seed genes. This directly affects the association scores at the gene level. In our cross-validation experiments, we selected seed genes as the union of those known as associated with top 10 diseases that have the highest phenotype similarity scores with the query diseases. This strategy seems to be effective. However, in a real application, the selection of seed genes may largely vary according to the disease under investigation. If the disease being studied has some known associated genes, it is effective to use these genes as seed genes. If no genes known to be associated with the disease of interest, we can still select genes associated with phenotypically similar diseases as seed genes. If the disease being studied has no known associated genes and the phenotype similarity information is not yet available for the disease, we can calculate phenotype similarities between the disease under investigation and other diseases based on the phenotypic presentation of the disease using our text mining technique in an online manner. Once phenotype similarities are obtained, we can perform the same procedure as described in the method section to obtain seed genes and identify causal variants for the disease of interest.

Besides, our approach can further be improved in the following directions. First, although nsSNVs in protein coding regions are the main focus in exome sequencing studies, SNVs in introns, various regulatory regions and intergenic regions occupy a majority in whole-genome sequencing studies. How to extend our method to analyze these non-coding variants in whole-genome sequencing studies will be our next focus. Second, besides SNVs, insertions, deletions and copy number variations also play an important role in human disease. How to extend our method to deal with these genetic variants is also an important direction. Third, our formulation depends on genomic data to infer causative variants, while a number of existing methods purely rely on genetic data to achieve this goal^50^. Although existing studies have demonstrated that the incorporation of a functional score into a statistical test could reasonably increase the power in detecting causative rare nonsynonymous variants, resulting in such methods as VTP^51^, VAAST^52^, PHIVE^53^ and Phen-Gen^54^, so far there is still no research systematically exploring the possibility of incorporating multiple functional scores of variants and association scores of genes into a statistical association test for rare variants. How to take advantage of such valuable functional scores to gain the power in the inference of disease rare variants is therefore another direction worth pursuing.

## Methods

### Data sources

We downloaded 25,559 causative nonsynonymous SNVs with annotation “Disease” and 38,910 neutral ones with annotation “Polymorphism” from the Swiss-Prot database^16^ (release 2014_01). We identified 1,984 genes containing the causative variants and extracted 2,799 associations between these genes and 2,511 diseases using Ensemble BioMart (http://www.ensembl.org/biomart). We mapped these diseases to their records in the OMIM database. We extracted 103,207 disease variants from the HGMD database (released in Feburary, 2014), with 24,925 of them also included in the Swiss-Prot database. Focusing on the 78,282 variants collected in HGMD only, we sampled 20,000 variants to obtain a test set of disease variants. We extracted 251,235 nonsynonymous exonic variants that occurred in 1,092 individuals from the 1000 Genomes Project.

We extracted 11 functionally damaging effect scores from our in-house database dbWGFP (http://bioinfo.au.tsinghua.edu.cn/jianglab/dbwgfp), which provided a comprehensive repository of annotations and functional predictions for more than 8.5 billion SNVs in the whole human genome. The functional scores are referred to as SIFT^11^, PolyPhen2^12^, LRT^29^, MutationTaster^30^, MutationAccessor^31^, MSRV^32^, GERP^33^, Phylop^34^, SiPhy^35^, CADD^36^ and SInBaD^37^. We derived 8 gene functional similarity matrices based on the gene expression^18^, gene ontology^55^, KEGG pathway^21^, protein sequence^56^, protein domain^22^, protein-protein interaction^19^, transcriptional regulation^39^ and microRNA regulation^40^. We derived a disease phenotype similarity matrix based on the OMIM and UMLS databases. The coverage of these data sources is shown in the Supplementary Materials (Table S1).

### Calculation of gene functional similarities

We calculated the gene expression similarity using genome-wide assays of 44,775 transcripts across 79 tissues^18^. To accomplish this, we represented a gene as a 79-dimensional vector, with a dimension corresponding to the expression value of the gene in a tissue. We then calculated the raw similarity between two genes as the absolute value of the Pearson’s correlation coefficient between corresponding vectors. Finally, we derived the gene expression similarity by applying an exponential transformation to the raw similarity, as


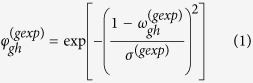


where 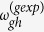 is the raw similarity between genes *g* and *h*, 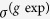 the standard deviation of all 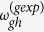 and 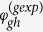 the gene expression similarity. Note that the exponential transformation amplifies the difference between large and small values, and thus conduces to filtering out weak similarities.

We focused on the biological process domain of the gene ontology^55^ and associated annotations for human genes^20^ to calculate the gene ontology similarity. We first extracted 25,616 gene ontology terms from the biological process domain and represented a gene as a vector, whose dimension was equal to the number of terms and whose elements denoted information contents of the terms. Here the information content of a term is defined as the negative logarithm of the relative occurrence frequency of the term in gene annotations. We then calculated the raw similarity between two genes as the cosine of the angle between the corresponding vectors and derived the gene ontology similarity according to the exponential transformation. A recent study has demonstrated that the direct application of the cosine measure, though simple per se, outperforms several other methods for calculating semantic similarities based on an ontology^57^.

We relied on the KEGG database^21^ to calculate the gene pathway similarity. From this database, we extracted 238 human pathways, with diseases-related ones filtered out to reduce the possible bias towards well-documented diseases. We represented a gene as a binary vector, whose dimension was equal to the number of pathways and whose elements denoted whether the gene was included in the pathways. We then calculated the raw similarity between two genes using the cosine measure and adopted the exponential transformation to obtain the gene pathway similarity.

We resorted to a sequence similarity network to calculate the protein sequence similarity. We downloaded from the Swiss-Prot database^56^ 20,274 sequences of human proteins and calculated their pairwise local alignments using the Smith-Waterman algorithm implemented in SSEARCH^58^. In order to enhance robustness, we constructed an undirected network to represent protein sequence similarity by connecting two proteins if their alignment e-value was less than a predefined threshold (10^−4^). We then calculated the shortest path distance, 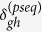, for every pair of proteins *g* and *h*, and used a linear transformation, 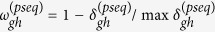, to derive the raw similarity 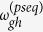. Finally, we adopted the exponential transformation to obtain the protein sequence similarity.

We relied on the Pfam database^22^ (Version 27.0) to calculate the protein domain similarity. We extracted from Pfam 14,831 protein domains and represented a human protein as a binary vector, whose dimension was equal to the number of domains and whose elements denote whether a domain existed in the protein. Similar to the derivation of the pathway similarity, we calculated raw similarity between two genes using the cosine measure and obtain the protein domain similarity according to the exponential transformation.

We relied on the STRING database^19^ (Version 9.1) to calculate protein network similarity. We extracted 403,514 interactions between 13,747 proteins from this database and constructed an undirected protein-protein interaction network. Similar to the derivation of sequence similarity, we calculated pairwise shortest path distances between proteins, used a linear transformation to obtain raw similarity between two genes, and calculated the protein network similarity according to the exponential transformation.

We relied on the TRANSFAC database^39^ to calculate gene transcriptional regulation similarity. We extracted from this database high confidence position weighted matrices (PWM) of 218 vertebrate transcription factors and identified their potential binding sites in promoter regions (1,000 basepairs upstream) of human genes using the program MATCH^39^. We then represented a gene as a 218-dimensional vector, with a dimension denoting the occurrence frequency of binding sites of the corresponding transcription factor in the promoter region of the gene. We then calculate raw similarity between two genes using the cosine measure and derived the transcriptional regulation similarity according to the exponential transformation.

We relied on the miRanda database to calculate gene microRNA regulation similarity. We collected 249 microRNAs and represented a gene as a binary vector, whose dimension was equal to the number of microRNAs and whose elements represented whether the gene is a target of a microRNA. We then calculate the raw similarity between two genes using the cosine measure and derived the microRNA regulation similarity according to the exponential transformation.

### Calculation of disease phenotype similarities

We calculated a disease phenotype similarity matrix using the text mining technique. Specifically, we first extracted 7,719 disease records from the OMIM database^26^ and split sentences in the text (TX) and clinical synopsis (CS) fields of these records into words. Then, we used the MetaMap program^59^ to map these words onto terms in UMLS and obtained 7,745 standardized terms for describing the diseases. Next, we represented a disease record as a vector, whose length was equal to the number of terms and whose elements corresponded to the TF-IDF values of the terms. Here, the TF-IDF value of a term was calculated as the product of the term frequency (TF, the relative occurrence frequency of the term in a disease record) and the inverse document frequency (IDF, the negative logarithm of the relative occurrence frequency of the records containing the term). Finally, we calculated the phenotype similarity between two diseases as the cosine of the angle between the corresponding vectors.

### Calculation of disease-gene association scores

We calculated the strength of association between a candidate gene and a query disease with gene similarities derived from different genomic data according to the guilt-by-association principle. To accomplish this, we first identified 10 diseases that were most phenotypically similar to the query disease according to the phenotype similarity matrix. Then, we collected genes known as associated with either the query disease or one of the 10 identified phenotypically similar diseases to obtain a set of seed genes. Finally, we calculated the summation of gene similarities (derived from e.g. PPI) between the candidate gene and all seed genes of the query disease to obtain the association score (e.g. PPI) between the candidate gene and the query disease. We performed the same procedure for gene similarities derived from different genomic data to obtain association scores for different genomic data.

### Prioritization via supervised learning

Given a query disease and a set of candidate variants, we assigned scores to the variants to indicate their strength of associations with the disease and then prioritized them accordingly. To accomplish this, we regarded a disease-variant pair as belonging to either a positive class (i.e., the variant is causative for the disease) or a negative category (i.e., the variant is not relevant to the disease), and we adopted a supervised learning method called the random forest to predict the likelihood that the disease-variant pair belonged to the positive class based on 19 numeric features. In the training phase, we collected from the Swiss-Prot database a set of labeled disease-variant relationships and trained a random forest model. In the test phase, we used the trained model to calculate prediction scores for candidate variants.

The numeric features includued 11 functional scores at the variant level and 8 association scores at the gene level. At the variant level, we queried the dbWGFP database for the variant in a disease-variant pair and extracted functionally damaging scores derived by such bioinformatics approaches as SIFT, PolyPhen2, LRT, MutationTaster, MutationAccessor, GERP, Phylop, Siphy, MSRV, SInBad and CADD. At the gene level, we calculated association scores between 7,719 OMIM diseases and 20,327 human genes under each of the 8 types of genomic data using the method detailed in the previous section. We then applied a linear transformation to adjust these scores according to two criteria: 1) a score should be in the interval of [0, 1] and 2) the larger a score, the stronger the evidence of functionally damaging. For SIFT and LRT, we firstly convert them by 1-SIFT and 1-LRT respectively. Then, we transform those scores (denoted as *f*) with formula 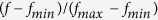, and the maximum and minimum values of those features are obtained from the whole database (e.g. dbWGFP). In the case that a feature is not available for a disease-variant pair, we assigned zeros to the missing data.

We obtained training data from the Swiss-Prot database. In the cross-validation experiments, we partitioned the 25,559 disease variants in this database into five positive subsets of almost equal sizes and the 38,910 neutral variants into two equal-sized negative subsets. In each validation run, we used one positive and one negative subsets for test and the remaining ones for training. We first identified disease-variant pairs in positive training subsets to obtain positive training data and collected diseases involved in. Then, we enumerated these diseases for every neutral variant in the negative training subset to obtain a pool of negative disease-variant pairs. Finally, we sampled from this pool the same number of pairs as positive ones to obtain negative training data. In the validation for rare variants, we used all disease variants except for the identified rare ones to generate positive training data and adopted the strategy detailed above to generate negative training data. In the spike-in validation for the 1000 Genomes Project, we adopted the strategy detailed above to generate positive training data, and used all neutral variants to generate negative training data. In real applications, we generated training data using all variants in the Swiss-Prot database. In all the experiments, we avoid information leakage by eliminating potential overlaps between diseases, genes and variants involved in the training and test phases.

As methods for comparison, we further implemented 4 widely used supervised learning approaches, including support vector machine (SVM), linear discriminant analysis (LDA), naïve Bayes and logistic regression. We adopted the same strategy as detailed above to obtain features and training data for these methods.

## Additional Information

**How to cite this article**: Wu, M. *et al.* Prioritization of Nonsynonymous Single Nucleotide Variants For Exome Sequencing Studies Via Integrative Learning on Multiple Genomic Data. *Sci. Rep.* **5**, 14955; doi: 10.1038/srep14955 (2015).

## Supplementary Material

Supplementary Information

## Figures and Tables

**Table 1:** Performance of different methods in cross-validation experiments.

	Neutral		Disease		Combined	
	MRR(%)	AUC(%)	MRR(%)	AUC(%)	MRR(%)	AUC(%)
eXtasy	7.77	92.20	43.48	56.50	25.87	74.12
SPRING	4.15	95.82	17.25	82.72	10.79	89.20
snvForest†	3.26	96.71	16.02	83.96	9.64	90.34
snvForest‡	3.37	96.60	15.97	84.00	9.76	90.23

^†^Results for the 19,742 variants extracted from the Swiss-Prot database.

^‡^Results for the 25,559 variants that eXtasy covers.

**Table 2:** Performance of individual features.

	Neutral			Disease		Combined
	MRR(%)	AUC(%)	MRR(%)	AUC(%)	MRR(%)	AUC(%)
SIFT	21.56	78.44	45.14	54.86	33.29	66.71
PolyPhen2	15.48	84.52	47.56	52.44	29.76	70.24
LRT	23.10	76.90	47.87	52.13	35.89	64.11
MutationTaster	27.43	72.57	53.06	46.94	40.42	59.58
MutationAccessor	17.59	82.41	52.13	47.87	32.99	67.01
GERP	23.73	76.27	47.19	52.81	35.62	64.38
Phylop	23.22	76.78	46.44	53.56	34.99	65.01
Siphy	21.14	78.86	46.77	53.23	34.15	65.85
MSRV	18.94	81.06	52.33	47.67	33.74	66.26
SInBad	17.16	82.84	53.00	47.00	35.33	64.67
CADD	15.29	84.71	54.49	45.51	35.16	64.84
Expression	34.07	65.93	37.13	62.87	35.63	64.37
GO	37.03	62.97	36.66	63.34	36.80	63.20
KEGG	23.38	76.62	35.46	64.54	29.60	70.40
miRNA	35.31	64.69	36.17	63.83	35.75	64.25
Pfam	31.04	68.96	31.43	68.57	31.24	68.76
Sequence	40.90	59.10	41.95	58.05	41.42	58.58
PPI	19.78	80.22	25.55	74.45	22.94	77.06
TSFC	33.81	66.19	35.25	64.75	34.54	65.46
All (snvForest)	3.37	96.60	15.97	84.00	9.76	90.23

Variant features are effective in distinguishing disease variants from neutral ones but are ineffectiveness in discriminating between disease variants. Gene features are medium effective in distinguishing disease variants from both neutral and disease controls. The integration of all the features yields much higher performance than any individual feature.

**Table 3:** Applications of snvForest to real exome sequencing studies.

PMID	Candidate Mutations	Funcional Mutations	Rank		*p*-value	
			Top 10	Top 20	Top 10	Top 20
23934111	192	30	8	13	5.3 × 10^−6^	9.4 × 10^−8^
23033978	77	16	5	10	2.8 × 10^−2^	5.5 × 10^−4^
23020937	126	17	5	7	4.1 × 10^−3^	6.4 × 10^−3^
