# Supplementary material for: Prioritization Of Nonsynonymous Single Nucleotide Variants For Exome Sequencing Studies Via Integrative Learning On Multiple Genomic Data
Source: Sci Rep. 2015 Oct 13;5:14955. doi: 10.1038/srep14955 (PMC4602202; doi:10.1038/srep14955)
Supplement: Supplementary Information [file srep14955-s1.doc]

# Supplementary Materials for

# Prioritization of nonsynonymous single nucleotide variants for exome sequencing studies via integrative learning on multiple genomic data

### Mengmeng Wu^1^, Jiaxin Wu^1^, Ting Chen^2,3^, Rui Jiang^1,4,§^

^1^ MOE Key Laboratory of Bioinformatics; Bioinformatics Division and Center for Synthetic & Systems Biology, TNLIST; Department of Automation, Tsinghua University, Beijing 100084, China

^2^ Department of Computer Science, Tsinghua University

^3^ Molecular and Computational Biology Program, University of Southern California

^4^ Department of Statistics, Stanford University

^§^ Corresponding author

Email addresses:

MW: [wmm13@mails.tsinghua.edu.cn](mailto:wmm13@mails.tsinghua.edu.cn)

JW: [wujiaxin0413@gmail.com](mailto:wujiaxin0413@gmail.com)

TC: [tingchen@tsinghua.edu.cn](mailto:tingchen@tsinghua.edu.cn)

RJ: [ruijiang@tsinghua.edu.cn](mailto:ruijiang@tsinghua.edu.cn)

# Supplementary Figures


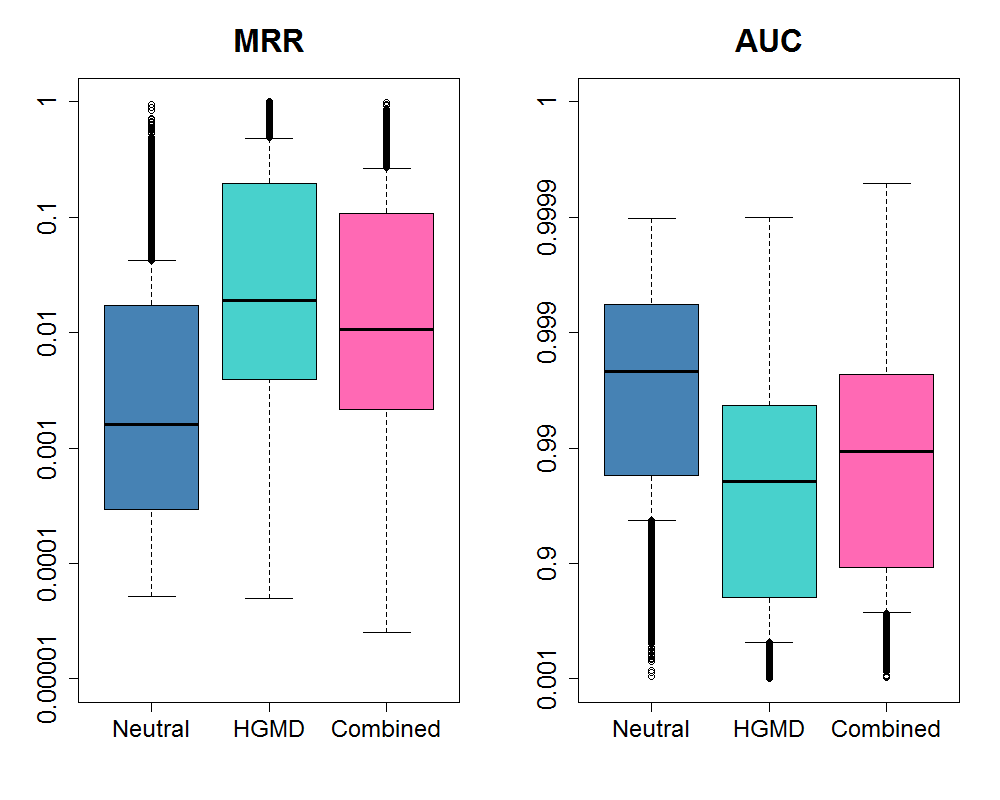


**Figure S1. Distributions of MRRs and AUCs for individual diseases in the cross-validation experiments.** The MRRs for individual diseases against the neutral, HGMD and combined test samples are mainly located in 0.1% ~ 1%, 1% ~10% and 0.1% ~ 10%, respectively, and the AUCs are mainly located in 99.0% ~ 99.9%, 90% ~99% and 90% ~ 99.9%, respectively. Specifically, the MRRs against neutral for 70.01% of diseases range from 0.1% to 1%, and the AUCs against neutral for 69.57% diseases range from 99.0% to 99.9%. These results demonstrate that our method can be effectively applied to most diseases.


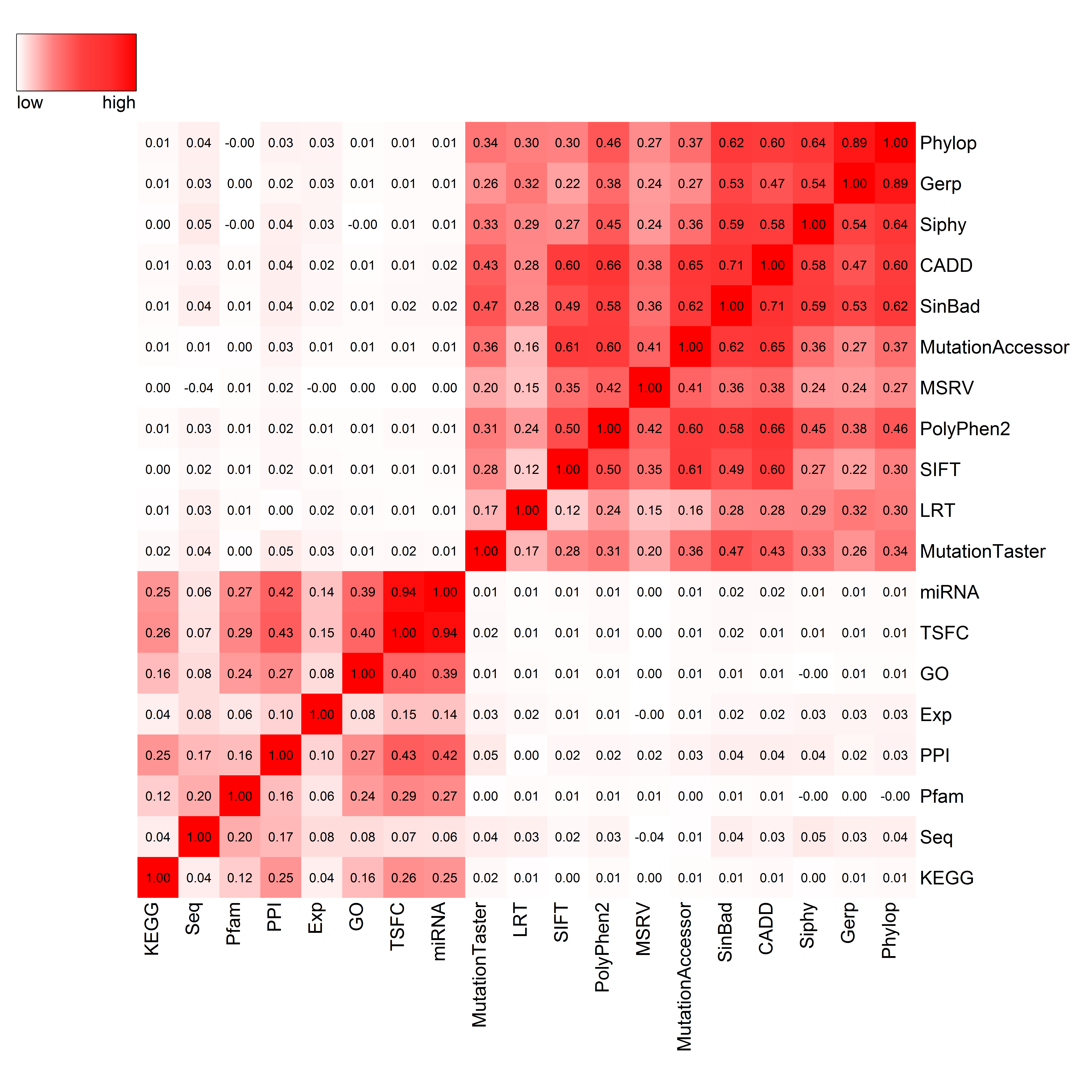


**Figure S2. Pairwise Pearson’s correlation coefficients between the features based on neutral variants.** In addition to the analysis in the main text, we further focused on the 38,910 neutral variants in the Swiss-Prot database to calculate pairwise Pearson’s correlation coefficients between the features. Results show that there still exist medium to strong positive correlations among the 11 functional scores at the variant level (top-right region), and the correlations between functional scores and association scores are also weak. Correlations among the association scores (bottom-left region), however, are much weaker than those calculated based on disease variants (Figure 5 in the main text), revealing that similarities between the gene hosting a neutral variant and seed genes of a query disease exhibit diversity among different data sources.


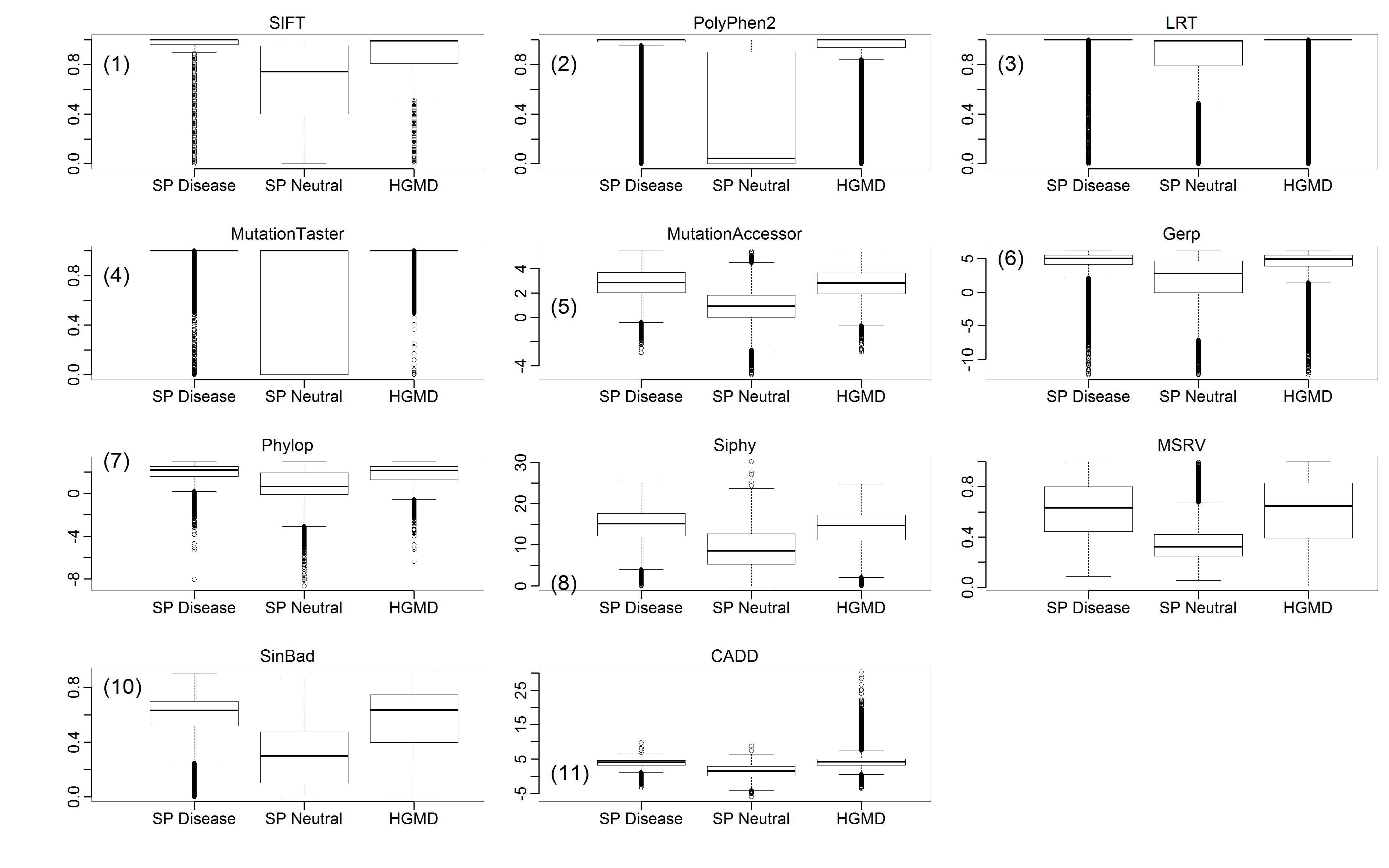


**Figure S3. Distributions of the 11 functional scores across different categories of variants.** SP Disease refers to the 25,559 variants annotated as “Disease” in the Swiss-Prot database. SP Neutral refers to the 38,910 variants annotated as “Polymorphism” in the Swiss-Prot database. HGMD refers to the 20,000 variants sampled from HGMD. The difference between distributions of neutral variants and disease variants is obvious, highlighting the power of these features for distinguishing between neutral and disease variants. On the other hand, distributions between Swiss-Prot disease variants and HGMD variants are not significantly different, explaining the ineffectiveness of these features in discriminating between disease variants.


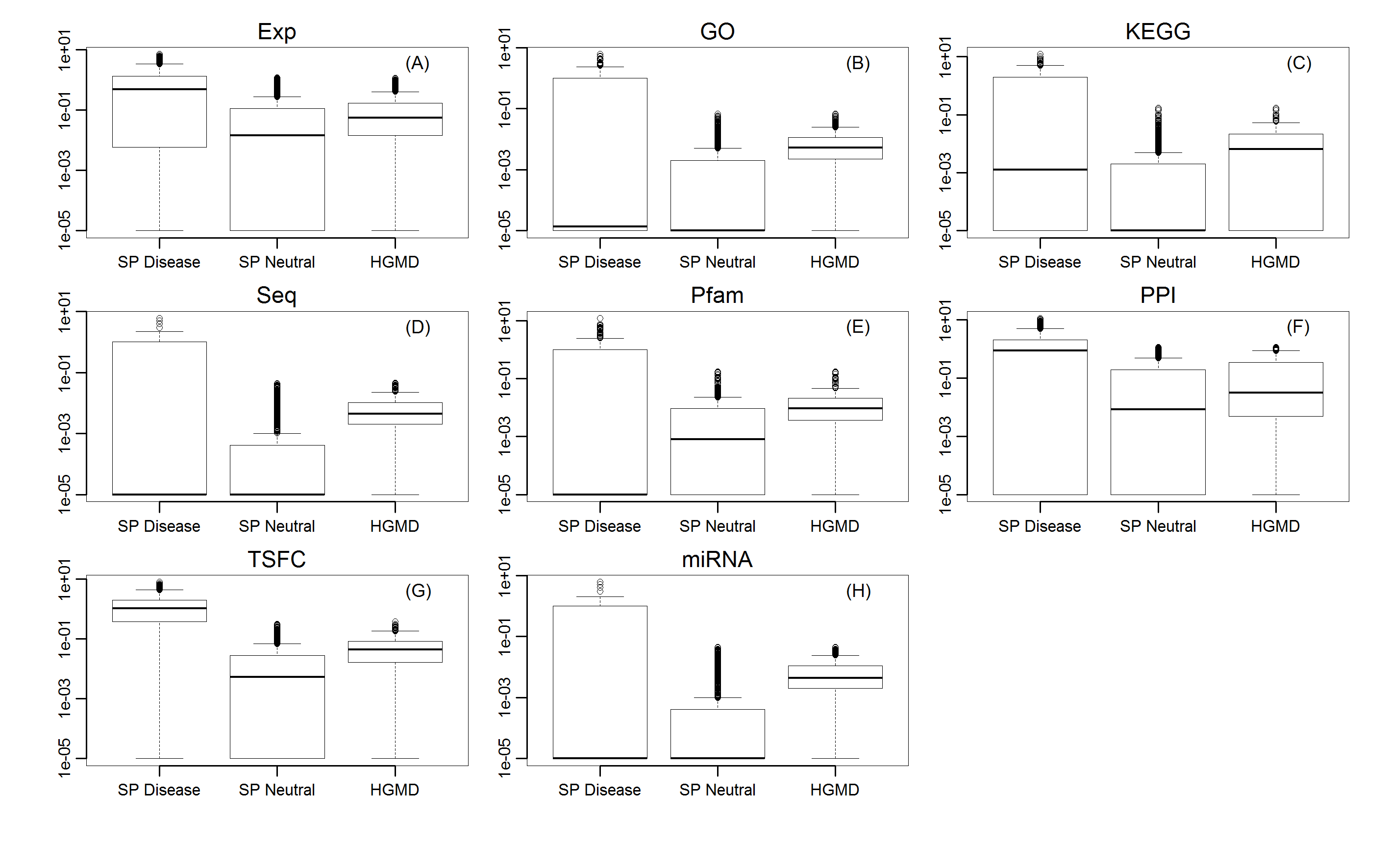


**Figure S4. Distributions of the 8 association scores across different categories of variants.** SP Disease refers to the 25,559 variants annotated as “Disease” in the Swiss-Prot database. SP Neutral refers to the 38,910 variants annotated as “Polymorphism” in the Swiss-Prot database. HGMD refers to the 20,000 variants sampled from HGMD. The difference between distributions of disease and neutral variants is obvious, highlighting the power of these features for distinguishing between neutral and disease variants. Distributions between variants causing a disease (SP Disease) and those causing other diseases (HGMD) are also different, suggesting the association scores have the ability to distinguish variants causing a specific type of disease from those responsible for other diseases.


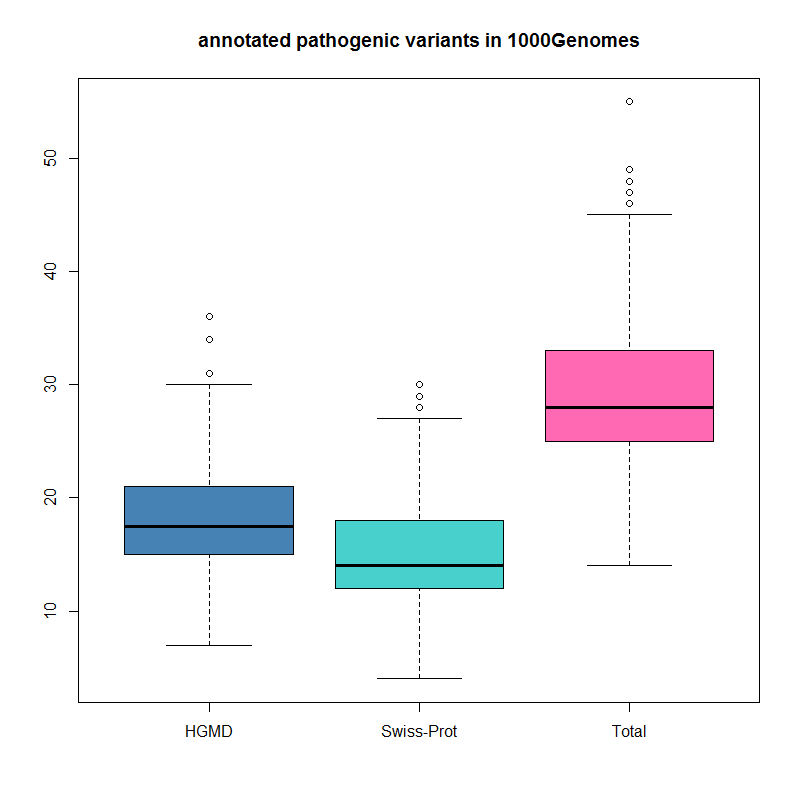


**Figure S5. Annotated pathogenic variants in the 1000 Genomes Project data.** Although individuals recruited by the 1000 Genomes Project are healthy, annotated causal variants from the Swiss-Prot and HGMD databases are still found. By mapping annotated disease variants into individual exomes, we find that on average about 30 pathogenic variants are present in an exome, revealing the importance of distinguishing variants causative for a query disease against those responsible for other diseases.


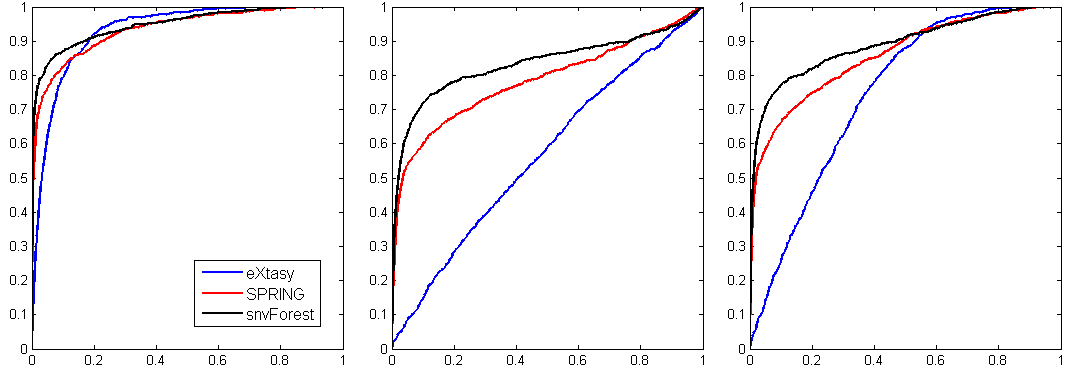


**Figure S6. Comparison of eXtasy, SPRING and snvForest with regard to the performance on rare variants prioritization.** The ROC curves illustrate the performance of eXtasy (Blue), SPRING (Red) and snvForest (Black) with regard to validation against the neutral (Left), disease (Middle) and combined (Right) test samples.

# Supplementary Tables

**Table S1. Coverage of the 11 functional scores and 8 association scores.** Coverage is calculated based on nonsynonymous single nucleotide variants in the following category: Swiss-Prot disease (25,559), Swiss-Prot neutral (38,910), HGMD disease (20,000) and the 1000 Genomes Project (251,235).

| **Features** | **Swiss-prot Disease (%)** | **Swiss-prot Neutral (%)** | **HGMD  Disease (%)** | **1000 Genomes Project (%)** |
| --- | --- | --- | --- | --- |
| SIFT | 95.99 | 96.62 | 93.00 | 56.18 |
| PolyPhen2 | 100.0 | 99.98 | 78.03 | 56.09 |
| LRT | 96.29 | 88.84 | 92.35 | 50.38 |
| MutationTaster | 99.98 | 99.90 | 99.89 | 61.72 |
| MutationAccessor | 98.92 | 98.34 | 77.12 | 56.48 |
| GERP | 100.0 | 99.96 | 99.98 | 100.0 |
| Phylop | 100.0 | 100.0 | 100.0 | 99.99 |
| Siphy | 99.87 | 99.46 | 99.84 | 99.95 |
| MSRV | 99.44 | 97.93 | 75.91 | 53.59 |
| SInBad | 100.0 | 100.0 | 100.0 | 61.98 |
| CADD | 100.0 | 100.0 | 100.0 | 100.0 |
| Expression | 93.21 | 72.13 | 94.37 | 69.14 |
| GO | 99.44 | 78.02 | 99.18 | 79.59 |
| KEGG | 65.55 | 34.85 | 68.16 | 34.25 |
| miRNA | 97.02 | 93.32 | 96.68 | 94.11 |
| Pfam | 73.50 | 76.66 | 81.40 | 91.07 |
| Sequence | 99.90 | 97.85 | 100.0 | 80.00 |
| PPI | 92.62 | 49.58 | 93.57 | 67.82 |
| TSFC | 100.0 | 100.0 | 100.0 | 100.0 |

**Table S2. Comparison of the performance of snvForest with existing methods.** For each type of the negative test samples (i.e., neutral, HGMD and combined), a pairwise one-sided Wilcoxon rank sum test is performed to check whether the median rank ratio of positive test variants for a method is smaller than that of another. Resulting raw *p*-values are then adjusted by Bonferroni correction. The significant *p*-values strongly support that the performance of these methods can be ordered from the highest to the lowest, as snvForest > SPRING > eXtasy.

|  | **Neutral** | **Disease** | **Combined** |
| --- | --- | --- | --- |
| snvForest *vs*. eXtasy | 0 | 0 | 0 |
| snvForest *vs*. SPRING | 2.03×10^-260^ | 2.93×10^-288^ | 1.11×10^-287^ |
| SPRING *vs*. eXtasy | 2.18×10^-127^ | 0 | 0 |

**Table S3.** **Comparison of the performance of different supervised learning methods****.** Cross-validation experiments, as detailed in section “Performance in cross-validation experiments” of the main text, are performed for each of the five machine learning methods, including snvForest, linear discriminant analysis (LDA), naïve Bayes (NB), logistic regression (LR) and support vector machine (SVM). Results suggest that the performance of these methods can be ordered from the highest to the lowest, as snvForest > SVM > LR > NB > LDA.

|  | **Neutral** | | **Disease** | | **Combined** | |
| --- | --- | --- | --- | --- | --- | --- |
|  | MRR | AUC | MRR | AUC | MRR | AUC |
| LDA | 6.97% | 93.00% | 27.63% | 72.34% | 17.44% | 82.55% |
| NB | 6.30% | 93.67% | 17.83% | 82.14% | 12.14% | 87.84% |
| LR | 4.62% | 95.35% | 17.76% | 82.21% | 11.28% | 88.70% |
| SVM | 4.64% | 95.33% | 17.34% | 82.63% | 11.08% | 88.91% |
| snvForest | **3.37%** | **96.60%** | **15.97%** | **84.00%** | **9.76%** | **90.23%** |

**Table S4. Comparison of the performance of different methods in validation experiments for rare variants.** Cross-validation experiments, as detailed in the section “Performance for rare variants” of the main text, are performed for each of the seven methods, including snvForest, linear discriminant analysis (LDA), naïve Bayes (NB), logistic regression (LR), support vector machine (SVM), SPRING and eXtasy. Results suggest that the performance of these methods for the neutral negative test sample can be ordered from the highest to the lowest, as snvForest > SPRING > eXtasy > SVM > LR > NB > LDA. For the HGMD and combined negative test samples, the performance of these methods can be ordered from the highest to the lowest, as snvForest > SPRING > SVM > LR > NB > LDA > eXtasy.

|  | **Neutral** | | **Disease** | | **Combined** | |
| --- | --- | --- | --- | --- | --- | --- |
|  | **MRR** | **AUC** | **MRR** | **AUC** | **MRR** | **AUC** |
| LDA | 14.10% | 85.89% | 39.19% | 60.81% | 26.82% | 73.18% |
| NB | 10.73% | 89.26% | 23.46% | 76.54% | 17.18% | 82.82% |
| LR | 8.17% | 91.83% | 22.35% | 77.64% | 15.35% | 84.64% |
| SVM | 7.90% | 92.09% | 21.28% | 78.82% | 14.68% | 85.32% |
| eXtasy | 7.68% | 92.31% | 46.27% | 53.72% | 27.24% | 72.75% |
| SPRING | 7.09% | 93.90% | 23.11% | 76.88% | 15.21% | 84.78% |
| snvForest | **6.24%** | **93.75%** | **19.14%** | **80.85%** | **12.78%** | **87.22%** |
